# Assessment of the Utility of Selected Inflammatory Markers in Correlation with Magnetic Resonance Enterography (MRE) Findings in the Diagnosis of Crohn’s Disease

**DOI:** 10.3390/biom15010116

**Published:** 2025-01-13

**Authors:** Justyna Lorenc-Góra, Dariusz Waniczek, Zenon P. Czuba, Mariusz Kryj, Zbigniew Lorenc, Małgorzata Muc-Wierzgoń

**Affiliations:** 1 Department of Imaging Diagnostics, St Barbara Regional Specialist Hospital No 5, 41-214 Sosnowiec, Poland; 2 Department of Imaging Diagnostics, Katowice Oncology Centre, 40-074 Katowice, Poland; 3 Department of Oncological Surgery, Faculty of Medical Sciences in Zabrze, Medical University of Silesia, 41-808 Katowice, Poland; dwaniczek@sum.edu.pl (D.W.);; 4 Department of Microbiology and Immunology, Faculty of Medical Sciences in Zabrze, Medical University of Silesia, 40-055 Katowice, Poland; 5 Department of General and Colorectal Surgery and Multiple Trauma, St Barbara Regional Specialist Hospital No 5, Medical University of Silesia, 40-055 Katowice, Poland; 6 Department of Internal Diseases Propaedeutics and Emergency Medicine, Faculty of Public Health in Bytom, Medical University of Silesia in Katowice, Piekarska 18, 44-902 Bytom, Poland

**Keywords:** Crohn’s Disease (CD), inflammatory markers, cytokines, calprotectin, magnetic resonance enterography (MRE), CDAI

## Abstract

Crohn’s Disease (CD) is a chronic inflammatory bowel disease affecting the gastrointestinal tract. The search continues for new markers for assessing the activity of CD. Among them, pro-inflammatory and anti-inflammatory cytokines appear promising. We performed the analysis of cytokine concentrations in blood serum using the Bio-Plex Multiplex system (Bio-Rad), and their correlations with radiological parameters were assessed by magnetic resonance enterography (MRE), and fecal calprotectin levels were measured quantitatively by ELISA and clinical evaluation according to the Crohn’s Disease Activity Index (CDAI). Our study found that measuring cytokine serum concentrations can be a valuable tool in the diagnosis and treatment of CD. Positive correlations were reported between contrast enhancement on DCE-MRE and the concentrations of PDGF-BB and RANTES. Also, a positive correlation was found between the delayed-phase of DCE and IL-10 concentration, a strong negative correlation between the delayed-phase of DCE and IL-12 concentration, and a strong positive correlation between the delayed-phase of DCE and RANTES concentrations. A strong positive correlation was also observed between the thickness of the intestinal wall on T2-weighted images and RANTES concentration. Therefore, concentrations of PDGF-BB, RANTES, IL-10 and IL-12 are promising markers of CD activity. The study also demonstrated significant correlations between the severity of disease activity assessed by the CDAI and the concentrations of IL-5, IL-8 and IL-9, as well as positive correlations between the levels of fecal calprotectin and the concentrations of IL-1RA and VEGF. Therefore, the levels of IL-5, IL-8, IL-9, VEGF and IL-1RA may be useful markers in the diagnosis and clinical assessment of disease activity.

## 1. Introduction

Crohn’s disease (CD) is a chronic condition with a complex and not fully understood etiology. It is classified as inflammatory bowel disease (IBD) [1,2,3,4]. Chronic inflammation in CD results from the impaired regulation of the immune response in individuals with genetic predispositions who are exposed to environmental factors [5]. Many tests are applied to diagnose and monitor CD treatment. The lack of biomarkers providing early diagnosis or defining the status of the pathology make an accurate assessment of the disease difficult [6]. They also include invasive procedures, such as colonoscopy with sample collection, which is the basic examination for establishing the final diagnosis and assessment of the disease severity. In the diagnostic process, especially in unclear clinical cases, laboratory findings and imaging diagnosis are also useful in differential diagnosis, the assessment of the extent and severity of the inflammatory process, the selection of optimal pharmacotherapy, or a decision on surgical treatment [7]. The search continues for additional diagnostic tools that could be widely available but not burdensome for patients [7,8,9]. Such tools can include the determination of cytokine levels. Acting as intercellular mediators, this diverse group of proteins affects the course of the inflammatory response by influencing the proliferation and stimulation of immune cells [10]. The impaired balance between pro- and anti-inflammatory cytokines plays a fundamental role in immune disorders underlying the inflammatory process in the intestinal wall [11]. Being markers of inflammation, cytokines may be crucial for the differential diagnosis of CD and may also be used to determine the disease severity/activity and to predict the course or risk of exacerbation in patients [4,7,12,13,14]. Among new methods of diagnostic imaging, magnetic resonance enterography (MRE) is of great importance since it is also a good tool for monitoring patients with CD. The examination is characterized by excellent tissue contrast and high spatial resolution, which allows precise information to be obtained about the morphology of the intestinal wall and changes outside the intestinal lumen. MRE is characterized by high sensitivity (>90%) in detecting active inflammation in the course of CD and is often useful in the preoperative assessment and making decisions about the radicality of treatment [15].

The aim of the study was to analyze the serum concentrations of selected inflammatory markers and their correlations with MRE parameters in patients with CD.

## 2. Materials and Methods

Forty-seven patients were enrolled in the study, including 24 patients with CD (study group; Group S) and 23 patients from the control group (Group C).

### 2.1. Characteristics of the Study and Control Groups

Group S consisted of 24 patients, including 14 women (58%) and ten men (42%) aged 22 to 78 years (mean age 45 years [SD ± 15.8], mean BMI 22.1 kg/m^2^ [SD ± 4.27]). Patients were either in remission or presented with varying degrees of inflammatory activity, which was determined by the Crohn’s Disease Activity Index (CDAI). Patients were admitted on an elective basis for surgical treatment of complications or on an expedited basis due to the exacerbation of CD or suspected sudden complications of the disease. The clinical characteristics and the basic laboratory findings of the study group are given in Table 1.

A commercial fecal calprotectin (FCAL) test was performed in all 24 patients from the study group (Group S). The concentration of calprotectin exceeded the normal range (50 μg/g) in 20 patients (83.33%). The results are given in Table 2.

Serological tests for the presence of ANCA anti-neutrophil cytoplasmic antibodies (perinuclear ANCA–pANCA and cytoplasmic ANCA–cANCA) were performed in most patients, and pANCA antibodies were found in 6 (20.8%) subjects, while cANCA antibodies were not found in any patient (Table 3).

MRE was performed in 17 out of 24 patients from Group S. The examinations were interpreted by board-certified gastrointestinal radiologists with appropriate experience in evaluating MRE images in patients with CD. Some patients also underwent additional examinations, such as colonoscopy, upper gastrointestinal endoscopy, rectoscopy, abdominal CT, abdominal X-ray and abdominal ultrasound. The diagnosis was confirmed in all patients by histopathological examination of postoperative material or samples obtained during colonoscopy (lesions in the colon or ileum).

Group C consisted of 23 patients who consented to the study, including 17 women (73.91%) and six men (26.09%) aged 50 to 85 years (mean age 65 years [SD ± 9.98]) with no history of IBD, cancer, or severe internal diseases. This group was recruited from patients eligible for surgery of varicose veins of the lower limbs or inguinal hernias. The characteristics of Group C are given in Table 4.

The analysis showed that BMI, age and body weight in Group S were significantly lower than in Group C, while height was higher in Group S. The analysis of histograms also showed that Group C did not include patients under 40 years of age, who constituted about 50% of Group S. No significant difference in the sex distribution was observed between the groups. The comparison of the groups is given in Table 5.

### 2.2. Determination of Serum Concentrations of Selected Markers of Inflammation

To determine selected markers of inflammation in blood serum, the Bio-Plex technique was applied (Bio-Rad, Laboratories, Hercules, CA, USA). This technique was chosen because of the possibility of simultaneous determination of many cytokines in a wide range of concentrations in a small sample volume (<50 µL). The Bio-PlexTM 200 System and Bio-Plex Manager™ software version 5.0 (Bio-Rad, Laboratories, Hercules, CA, USA) were used to read the data. Measurements were carried out in accordance with the manufacturer’s instructions. The Bio-Plex method is based on a set of magnetic beads that differ in the shade of red that corresponds to the specificity of the antibodies covering them that are directed to specific cytokines. Each shade of red corresponds to a specific cytokine. During incubation, bead-bound cytokines were detected using biotinylated antibodies stained with a streptavidin-phycoerythrin complex. After each step of the reaction, a 96-well microplate containing the bead mixture was rinsed using an ELx 50 magnetic washer (BioTek Instruments, Winooski, VT, USA). Quantitative measurements of cytokine concentrations were made based on the obtained median fluorescence of individual groups of beads for a given sample and the curves obtained for a series of dilutions of appropriate standards using the company software. The obtained cytokine concentration results were then exported to Excel and analyzed using other programs.

The serum cytokine concentrations were measured at least twice. For the final statistical calculations, the mean of the determinations was considered. If the standard deviation (SD) from the sample exceeded the assumed values, the sample was determined again. The software automatically determined the minimum and maximum detection levels of the analyzed analytes based on the data. If the concentration of the analyte was below the lower limit of quantification (LLOQ), its value was assumed to be equal to the LLOQ for a given analyte (cytokine). The following cytokine panel was determined in the samples: IL-1β, IL-1RA, IL-2, IL-4, IL-5, IL-6, IL-7, IL-8, IL-9, IL-10, IL-12 (p70), IL-13, IL-15, IL-17A, eotaxin, FGF-basic, G-CSF, GM-CSF, IFN-γ, IP-10, MCP-1 (MCAF), MIP-1α, MIP-1β, PDGF-BB, RANTES, TNF-α and VEGF.

### 2.3. MRE in the Study Group

In Group S, MRE examinations were finally performed in 17 out of 24 patients (eight women—47.1% and nine men—52.9%). The remaining seven patients were disqualified from the study due to clinically diagnosed bowel obstruction confirmed by CT scans and the need for urgent surgery, while some patients refused the examination or MRE was discontinued due to the patient’s discomfort.

**Figure 1:**
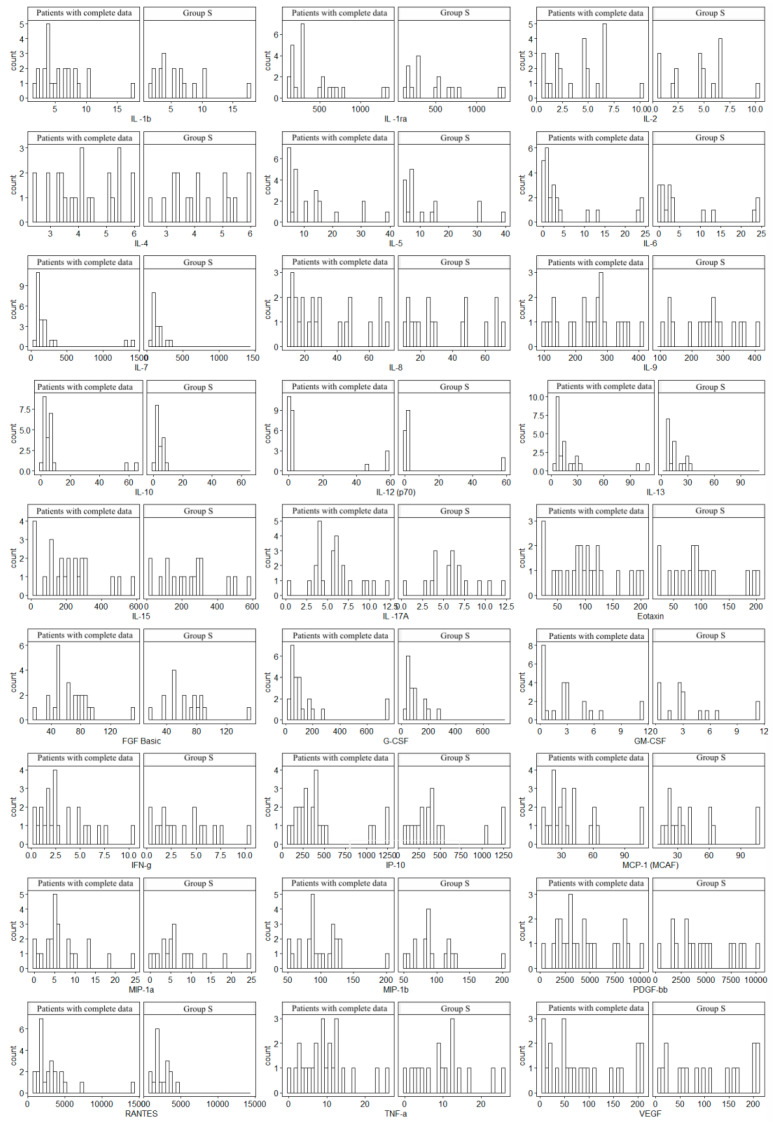
Comparison of cytokine concentration distributions of 17 patients with complete data (including MRE) versus Group S (the total group of 24 patients). The study was approved by the Bioethics Committee of the Medical University of Silesia (Resolution No. PCN/0022/KB1/98/I/18/19 of 12 November 2019).

Since not all patients underwent MRE, the histogram analysis was used to assess the consistency of the obtained data (17 patients were examined in relation to the entire Group S). In all cases, similar distributions of cytokine concentrations were observed, which supports the homogeneity of the groups. Histograms are given in Figure 1.

### 2.4. Statistical Analysis

Power analysis was performed using the PWR package for R in the RStudio environment. Assessment of the normality of the distribution for quantitative variables was performed using the Shapiro–Wilk test. In the case of IL-15 and FGF, one missing datum was observed in each case in the study group. The missing data were replaced with the values matched based on the random forest algorithm from the MissForest library for the R language. The normalized root mean square error (NRMSE) was 0.02. Quantitative data are presented as the median with a quartile range due to the low sample size and failure to meet the normal distribution. A nonparametric Mann–Whitney U test was used to compare the groups. The relationships between the variables were analyzed using the Spearman correlation coefficient. Qualitative variables are presented as the percentage of cases in which a given feature was found. The values of *p* < 0.05 were considered statistically significant. The results were developed in R using the RStudio environment (RStudio Team 2020; RStudio: Integrated Development for R. RStudio, PBC, Boston, MA, USA, URL http://www.rstudio.com/, accessed on 22 November 2024 and Microsoft Excel 365). The principal components method with Varimax rotation was used for PCA analysis. Two principal components were obtained and used as variables in further analysis.

## 3. Results

For clarity, the results of serum inflammatory biomarker concentrations were divided into two subsets, i.e., the results of interleukins (ILs) and other cytokines (Table 6 and Table 7). Except for IL-5, IL-12 and IL-15, the levels of most cytokines and soluble receptor antagonist (IL-1RA) were significantly higher in Group S than in Group C. Significantly higher concentrations of eotaxin, IFN-γ, IP-10, MCP-1, MIP-1β, PDGF-BB, RANTES, TNF-α and VEGF in Group S were reported compared to Group C. The concentrations of FGF-Basic, G-CSF, GM-CSF and MIP-1α did not differ significantly.

**Figure 2:**
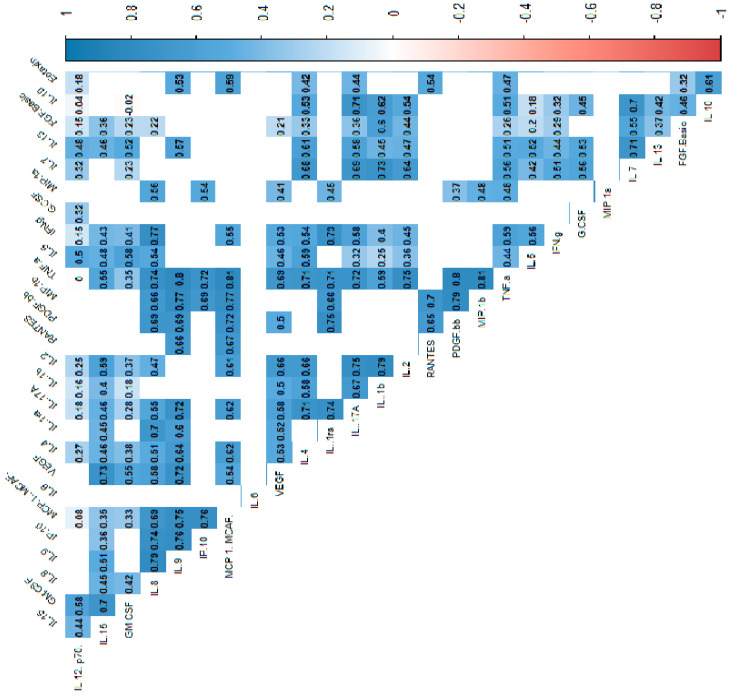
Correlation values of the cytokines and IL-1RA in the control and study groups. The data are presented as a heat map with hierarchical grouping. The figure shows only statistically significant correlations at *p* < 0.05. The numbers inside the fields represent Spearman’s correlation coefficients.

**Figure 3:**
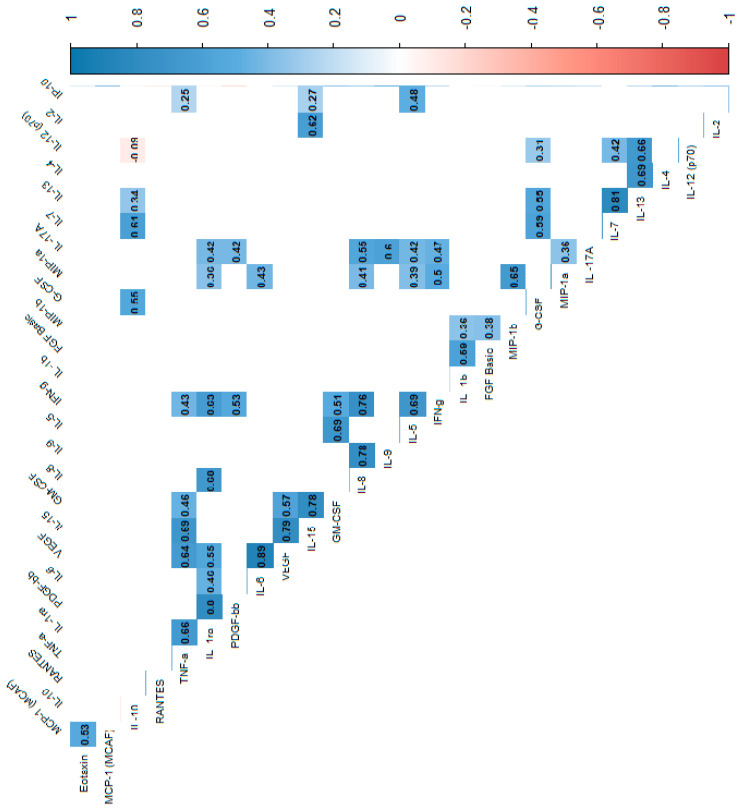
Correlation values of the cytokines and IL-1RA in the study group. The data are presented as a heat map with hierarchical grouping. The figure shows only statistically significant correlations at *p* < 0.05. The numbers inside the fields represent Spearman’s correlation coefficients.

**Figure 4:**
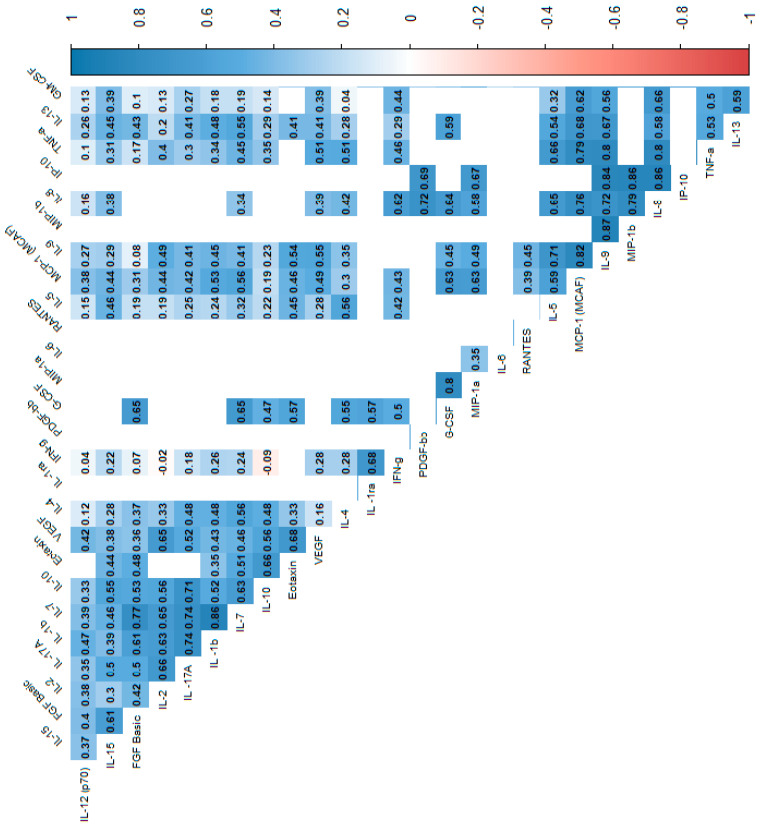
Correlation values of the cytokines and IL-1RA in the control group. The data are presented as a heat map with hierarchical grouping. The figure shows only statistically significant correlations at *p* < 0.05. The numbers inside the fields represent Spearman’s correlation coefficients.

In the power analysis performed in most variables quite high calculated power (>0.6) was achieved. Next, we analyzed the correlations between cytokines, which indicated that most of the cytokine concentrations were positively correlated. After stratification into groups, no significant trend reversal was found in the form of a change in the direction of correlation from strongly positive to strongly negative, which suggests no significant effect in the mechanism of cytokine action between the groups. Cytokine correlations are shown in the heat maps (Figure 2, Figure 3 and Figure 4).

Spearman’s correlation coefficient was used to analyze the relationships between the concentrations of all cytokines and the following radiological parameters (MRE):

- Enhancement of the intestinal wall assessed using DCE;
- Enhancement of the intestinal wall assessed using delayed-phase of DCE;
- Thickness of the intestinal wall on T2 images;
- Diffusion restriction assessed using apparent diffusion coefficient (ADC) maps (mm/s).

The analysis showed a positive correlation between contrast enhancement on DCE and the concentrations of PDGF-BB and RANTES. A positive correlation was also observed between contrast enhancement in the delayed phase and IL-10 concentration. A strong negative correlation was found between contrast enhancement in the delayed phase and IL-12 concentration. A strong positive correlation was reported between contrast enhancement in the delayed phase and RANTES concentrations. A strong positive correlation was also observed between the thickness of the intestinal wall on T2 images and the concentration of RANTES, and a tendency to a positive correlation between the thickness of the intestinal wall and the concentrations of IL-10. However, no significant relationships were observed between diffusion restriction and the cytokines.

**Figure 5:**
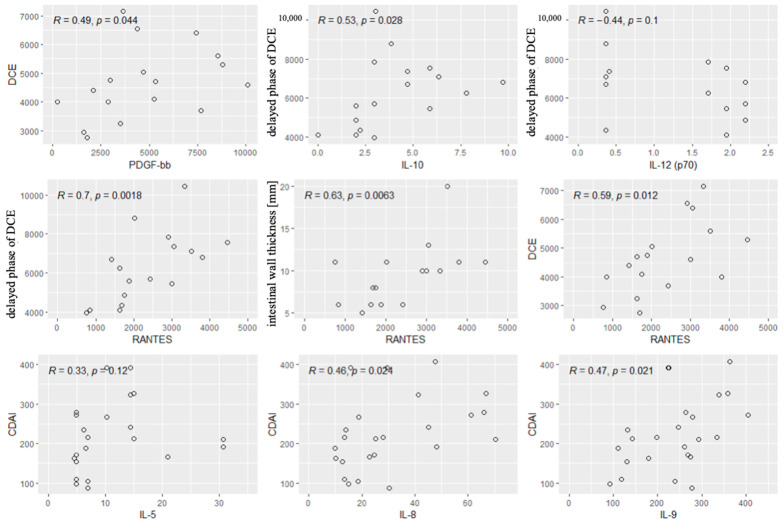
Significant correlations between radiological parameters and the CDAI and the cytokines. R—Spearman’s correlation coefficient, *p*—*p*-value (test probability).

Next, we performed the analysis of the correlations between the values of CDAI and the cytokines and radiological parameters. Significant correlations were found between the CDAI values and the levels of IL5, IL-8 and IL-9. Other cytokines and all radiological parameters did not correlate significantly with the CDAI. Significant correlations are shown in Figure 5.

**Figure 6:**
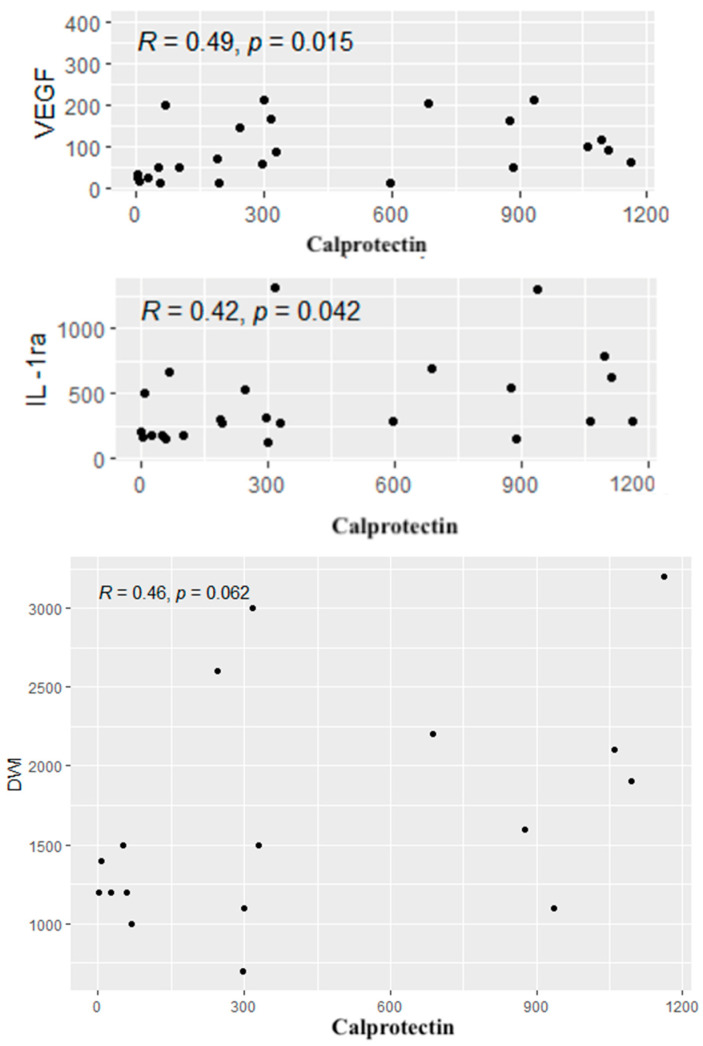
Significant correlations between the cytokines, radiological parameters and the level of calprotectin. R—Spearman’s correlation coefficient, *p*—*p*-value (test probability).

A similar analysis was performed between calprotectin concentrations and the cytokines, CDAI values and radiological parameters. Positive correlations were found between calprotectin concentrations and the levels of IL-1RA and VEGF. In addition, a tendency to a positive correlation was observed between the level of calprotectin and the diffusion restriction on DWI (*p* = 0.06). Significant correlations are given in Figure 6.

## 4. Discussion and Conclusions

The search for new diagnostic and therapeutic methods in CD is very important because this chronic and incurable gastrointestinal disease characterized by alternating periods of exacerbation and remission mainly affects young people. In this group of patients, absorption disorders often occur and lead to weight loss. In addition, patients with active disease often limit their food intake due to abdominal pain, nausea and discomfort after the consumption of certain products, which leads to an insufficient supply of iron and malnutrition. Similarly, our patients were mainly young subjects who significantly differed in age and weight from the controls.

Cytokines play a crucial role in the pathogenesis, diagnosis and prevention of Crohn’s disease (CD) [10,11,12,13,14]. Numerous authors and researchers have extensively discussed the role of cytokines in Crohn’s disease. These studies emphasize primarily the importance of cytokines such as TNF-α, IL-6, IL-10, IL-12, IL-17 and IL-23 in the disease’s pathogenesis, particularly their roles in promoting or regulating inflammation. In our study, we included the analysis of variability in the concentrations of 28 cytokines–IL-1β, IL-1RA, IL-2, IL-4, IL-5, IL-6, IL-7, IL-8, IL-9, IL-10, IL-12 (p70), IL-13, IL-15, IL-17A, eotaxin, FGF-basic, G-CSF, GM-CSF, IFN-γ, IP-10, MCP-1 (MCAF), MIP-1α, MIP-1β, PDGF-BB, RANTES, TNF-α and VEGF in correlations with radiological parameters assessed by magnetic resonance enterography (MRE), fecal calprotectin (FCAL) levels and clinical evaluation according to the Crohn’s Disease Activity Index (CDAI). There are no similar studies in the available literature.

It has long been known that the imbalance between pro-inflammatory cytokines is responsible for disorders of intestinal immune mechanisms in IBD [(IL-1(IL-1α, IL-1β), IL-2, IL-6, IL-8, IL-12, IL -17, IL-23, TNF-α, IFN-γ)] and anti-inflammatory cytokines (IL-4, IL-10, IL-11, IL-13). Additionally, particular interest has been aroused by the specific type of response in CD and UC, which determines the cytokine profile in the pathogenesis of these diseases. It has been proven that the cellular response dominates in CD, hence the main cytokines responsible for the development of the inflammatory reaction are those released by activated effector lymphocytes (Th1 and Th17 subpopulations). Th1 lymphocytes produce mainly IL-2, IFN-γ and TNF-α, while Th17 lymphocytes mainly produce IL-17 and IL-22. It is also known that there is an imbalance between the above-mentioned cytokines produced by effector T lymphocytes (activated subpopulations of Th1 and Th17 lymphocytes) and cytokines produced by Treg (regulatory) lymphocytes, i.e., IL-10 and TGF, which are responsible for inhibiting excessive inflammatory response. In UC, however, there is an increased expression of a subpopulation of Th2 lymphocytes, which results in the activation of NKT cells (a subpopulation of lymphocytes that have the characteristics of both lymphocytes and NK cells), resulting in the production mainly of IL-5 and IL-13 [16].

Martinez-Fiero et al. [17] analysed the 27 protein profiles of serum from 53 participants (23 UC, 11 CD, and 19 controls) according to pharmacologic therapy and laboratory and clinical findings in IBD. In this work, elevated serum levels of G-CSF, IL-1Ra and PDGF-BB were associated with IBD endoscopic activity. PDGF-BB also correlated with serum levels of IL-4, IL-8, IL-15 and IL-17, reflecting the diversity of molecular pathways that operate in parallel in IBD and the complexity of interactions between them. Also, their result showed that, compared with controls serum IL-15, eotaxin, MCP-1 and PDGF-BB levels were higher in CD but not in UC patients, which is partially consistent with our research, where significantly higher concentrations of eotaxin, MCP-1, PDGF-BB among others: IFN-γ, IP-10, MIP-1β, RANTES, TNF-α and VEGF in Group S were reported compared to Group C. Interestingly, in our study, serum IL-15 was not significantly higher in Group S than in Group C, but actually, except for IL-5, IL-12 and IL-15, the levels of most cytokines and soluble receptor antagonist (IL-1RA) were significantly higher in Group S than in Group C.

Acute phase protein (CRP) and FCAL are commonly used markers of inflammation in CD, which allows the monitoring of the disease course and response to treatment. CRP is produced in the liver in response to inflammation induced by IL-1, IL-6 and TNF-α. A highly significant positive correlation was observed between IFN-γ and CRP, and FCAL levels (*p* < 0.01). Moreover, FCAL levels showed a significant positive correlation with Th1- and Th17-associated cytokines (IL-6, TNF-β, SAA and IL-17A) [18]. Despite certain limitations (lack of specificity), it is useful in assessing disease activity. In correlation with other markers, it allows the course of CD and response to treatment to be monitored. Fecal calprotectin, on the other hand, is a protein formed in the liver with the participation of cytokines, whose blood concentration changes as a result of response to inflammation. Fecal calprotectin is commonly used as a biomarker in CD and in differentiating CD from irritable bowel syndrome (IBS), which has a different pathogenesis despite similar symptoms. It has been proven that increasing FCAL levels in routine tests predicts disease progression regardless of the presence of symptoms and location of CD. Stable normal FCAL levels are also considered a positive prognostic marker for maintaining clinical remission. Other studies have suggested that FCAL levels may be a non-invasive marker of mucosal healing. Research is conducted on other biomarkers that can potentially be used in clinical decision-making [7,19,20,21,22]. Recent advances have also demonstrated that the combination of MRE with calprotectin has improved the diagnostic accuracy and monitoring of CD. According to Jones and colleagues [23], Fecal calprotectin correlates well with the MRE assessment of ileal CD, and MRE parameters are associated with long-term biologic- and surgery-free remission. Other authors [24,25] suggest that inflammation detected by MRE is often associated with low endoscopic healing index and FCAL in similar proportions.

In recent years, cytokines have been considered promising biomarkers since long-term follow-ups have shown that this group of proteins plays a vital role in the pathogenesis of CD, particularly with regard to the disproportion between pro-inflammatory and anti-inflammatory cytokines [7,19,26,27,28]. The assessment of their concentrations can be useful for establishing an appropriate diagnosis and assessing the severity of disease activity. Additionally, it can be a potential prognostic tool for predicting the course of the disease and facilitating the development of new therapeutic methods, including biological treatment [29,30,31]. According to the literature, it is clinically more useful to determine several biomarkers at the same time, and their combined assessment can be an effective tool for monitoring patients. It might replace invasive testing in the future or reduce the number of tests. Among the promising cytokines, interleukin 6 (IL-6) is crucial. It has a pro-inflammatory activity. Many authors, such as Nikolaus et al. [31], found elevated serum levels of IL-6 in patients with active CD. Similarly, in the group of our patients, the concentrations of IL-6 were significantly higher compared to the controls. There are also reports that the assessment of IL-6 can be used as a biomarker in predicting response to biological therapy, although study results are inconclusive [28,32]. However, this is an interesting point for further research since IL-6 has been the focus of attention for many years (the possibility of targeted anti-IL-6 therapy in patients with severe CD) [33]. Cytokine regulatory networks have important implications for the development and progression of the disease [31], which is demonstrated by the failure of IL-10 in treating CD. Initially, IL-10 supplementation had beneficial effects in mouse models, and clinical trials showed that recombinant IL-10 was safe and well tolerated in humans. Unfortunately, the effect of clinical trials in humans was disappointing. Based on current research, IL-10 supplementation cannot be a universal treatment method in CD, but it can provide the expected effect in a well-selected small group of patients [34]. In our subjects, the levels of IL-10 were significantly higher compared to the control group, which shows how important it is to determine the concentration of cytokines because, in these patients, IL-10 supplementation would certainly not be beneficial and could even exacerbate the already ongoing inflammation.

Another interesting biomarker is RANTES, a chemokine synthesized by T lymphocytes, which has a pro-inflammatory effect. Ansari et al. [35] found that the concentrations of RANTES were elevated in CD. However, significantly higher levels of RANTES were noted in ulcerative colitis (UC) compared to CD. Therefore, it seems important to consider the concentration of RANTES in differential diagnosis (UC vs. CD) and when inflammatory changes in the intestinal wall are suspected. This study showed correlations between RANTES and MRE parameters indicative of inflammation in the intestinal wall, such as wall thickening and contrast enhancement on DCE. A similar correlation was found between platelet-derived growth factor (PDGF) and the enhancement of the intestinal wall on DCE, which is in line with the literature data related to increased PDGF levels in patients with active CD [36]. These correlations indicate that it is worth considering the assessment of PDGF and RANTES levels in a group of patients with suspected inflammatory bowel changes. The concentration of RANTES seems crucial in correlation with the contrast enhancement of the intestinal wall and its thickness. As we know, the clinical severity of the disease does not always correlate with the symptoms reported by patients and the CDAI. It may be useful to determine disease activity using other non-invasive markers that can precede the clinical manifestation of the disease and the symptoms reported by patients. Currently, scientists are focusing on defining the goal of therapy and then striving to achieve it in accordance with the “treat to target” strategy. This treatment method aims to reduce the activity and inhibit the progression of the disease based on the assessment of the activity of the inflammatory process (including through the assessment of biomarkers and endoscopic examinations), and not on the basis of the symptoms. This strategy involves regularly assessing disease activity and then adapting and modifying therapy to achieve the intended clinical effect. Thus, cytokines, such as RANTES and PDGF-BB, can support the radiological monitoring of CD, allowing for a more comprehensive assessment of patients. Confirmation comes from the latest research by Fang et al. [37] which indicates that PDGF-BB levels in mucosal tissue may be independent risk factors for active IBD. Moreover, PDGF-BB levels were positively correlated with M1 macrophage markers.

Nevertheless, long-term research is still needed to determine the most appropriate goals of selected therapeutic strategies and their final effects [30].

The literature shows the correlations of individual biomarkers with disease activity measured using the CDAI. To date, there has not been sufficiently strong evidence that the determination of the level of a single interleukin could be a sufficient tool to assess CD activity. However, determining several biomarkers is more clinically useful and reliable, and their combined assessment can be an effective tool for monitoring patients. Bourgonje et al. [32] proved that the combined assessment of four biomarkers (serum amyloid A, IL-6, IL-8 and eotaxin 1) reliably predicted disease activity in correlation with endoscopic assessment. In another study, Słowińska-Solnica et al. [38] showed that the combined assessment of biomarkers offered better results in the diagnosis of CD than each of these markers alone. The best results were obtained by assessing IL-23, CRP, IL-6 and IL-17 together. Also, more accurate results were obtained for the combined assessment of IL-6 and CRP than for each biomarker separately. In addition, there have been some findings that the concentration of IL-6 correlated well with the CDAI, and its concentration could predict a clinical response during biological therapy [28,39]. Surprisingly, no correlation was found between the CDAI and IL-6 levels in the study group. However, correlations were reported between IL5, IL-8 and IL-9 levels and the CDAI, which could contribute to a broader use of these interleukins to monitor patients with CD. IL-5 is a chemotactic agent for the survival and activation of eosinophils, which play a role in mucosal immunity [35]. During active inflammation, eosinophils increase IL-4, IL-5 and IL-13 expression, indicating a shift to the Th2 response. In response to IL-5, eosinophils are released into the peripheral circulation, after which they can migrate to the gastrointestinal tract after the binding of chemoattractant molecules:CCL11, eotaxin1, CCL24–eotaxin2, CCL26 -eotaxin3, CCL5-regulated upon activation, normal T cell expressed and secreted (RANTES), CCL7–MCP-3 and CCL13 (MCP-4) [40]. Eotaxines are chemokines that bind to eosinophils’ surface receptor CCR3, resulting in eosinophil migration and homing in the lamina propria of the bowel mucosa [41]. In some cases of Crohn’s disease, eosinophil infiltration is observed, particularly during inflammation or in areas of active disease. Eosinophils in surgical specimens of resected ileum of CD patients have been linked to an increased risk of early recurrence, while peripheral blood eosinophilia has been shown to predict clinically active disease in pediatric CD patients and has been associated with increased disease severity [42,43].

In the case of IL-8, next to the demonstrated correlation with the CDAI, an additional advantage is its high accuracy in differentiating patients with IBD from IBS. IL-8 (also known as CXCL8) is a pro-inflammatory chemokine primarily involved in the recruitment and activation of neutrophils. The synthesis of IL-8 is strongly stimulated by IL1-β, TNF-α, and bacterial lipopolysaccharides (LPS) [44]. It plays a significant role in the innate immune response and is produced by various cells, including macrophages, epithelial cells and endothelial cells. IL-8 is a potent chemoattractant for neutrophils, which are a hallmark of acute inflammation in Crohn’s disease. Studies have shown that IL-8 levels are elevated in the serum, stool and mucosal tissues of patients with active Crohn’s disease. IL-8 is thought to perpetuate the inflammatory cycle by recruiting more neutrophils and enhancing the release of reactive oxygen species (ROS) and proteolytic enzymes [45].

IL-9 is a cytokine primarily associated with the Th9 subset of T-helper cells. It has pleiotropic effects, influencing mast cells, eosinophils and epithelial cells, and is involved in allergic responses and mucosal immunity [46]. IL-9 levels are elevated in the intestinal tissues of patients with Crohn’s disease. It promotes the production of other pro-inflammatory cytokines (e.g., TNF-α, IL-6) and chemokines that amplify the inflammatory response. By activating mast cells and fibroblasts, IL-9 contributes to extracellular matrix remodeling and fibrotic changes [47]. IL-5, IL-8 and IL-9 contribute to Crohn’s disease through distinct yet interconnected pathways involving immune cell recruitment, barrier dysfunction and chronic inflammation. Their roles vary across disease stages and phenotypes, with potential synergies in amplifying inflammation and fibrosis. Understanding these relationships could guide more targeted therapeutic strategies [47].

Scaioli et al. [47] focused on the correlation between the calprotectin concentration in the stool with the CDAI. Their study showed that patients with a CDAI value from 100 to 150 had an increased probability of increased fecal calprotectin levels and asymptomatic mucosal inflammation. Their study also found that asymptomatic patients with a CDAI = 120 had a 60% chance of having endoscopically/histologically evident active disease. Therefore, the authors of the study showed that it could be useful to analyze a group of patients with a CDAI value ranging from 100 to 150. Our study showed positive correlations of calprotectin levels with selected cytokine concentrations (IL-1RA, VEGF), which confirms the importance of calprotectin as a biomarker of inflammation in CD. However, we found no correlation between calprotectin concentrations and the CDAI. IL-1RA is an IL-1R receptor antagonist mainly produced together with IL-1 to prevent overstimulation and inflammatory response. The correlation we demonstrated suggests that it is worth considering the assessment of IL-1RA in CD, although the IL-1RA/IL-1 ratio is probably a more reliable factor. Casini Raggi et al. [48] suggested that the impaired imbalance between Il-1RA and IL-1 could indicate a disturbance in homeostasis and the ratio was strongly correlated with disease severity. This finding is significant since it has also been previously reported that the concentration of IL-1RA needed to be about 100 times higher compared to IL-1 to effectively inhibit IL-1 signaling [49,50]. In our opinion, it was essential to demonstrate a correlation between the growth factor involved in the formation of the blood vessel network, such as VEGF, and the concentration of calprotectin. VEGF is a protein considered to be the most potent factor promoting angiogenesis. One paper showed that serum VEGF levels correlated well with the clinical picture of patients and the parameter could also be used to predict response to anti-TNF-α therapy. Eder et al. (2015) [51] suggested that VEGF could be an additional marker of CD activity and the assessment of its concentration in blood serum could be useful in optimizing the treatment with anti-TNF-α and in selecting patients who could benefit from this therapy. Therefore, our demonstration of the correlation between VEGF and fecal calprotectin levels is further evidence of the possible use of this growth factor as a biomarker in CD. When monitoring patients with CD, attention should be paid to the course of the disease, its activity and endoscopic surveillance since the long-term course of the disease increases the risk of carcinogenesis, and patients with IBD are also affected with colorectal cancer, usually at a younger age than the general population [52]. It is believed that chronic inflammation in the intestinal wall is one of the crucial factors in the pathogenesis of colorectal cancer and adenocarcinoma of the small intestine. It has been proven that the ongoing inflammatory process and the imbalance between the initiation and inhibition of the inflammatory response affected neoplastic transformation, cell proliferation, angiogenesis and metastasis. It is also believed that many of the interleukins associated with CD, including IFN-γ, IL-1, IL-2, IL-6, IL-8 and IL-17, play an essential role in carcinogenesis [53,54,55,56]. Further research is warranted on the mechanism of action of individual cytokines and their importance in CD since they allow the improvement of existing therapies and the creation of new ways for regulating cytokine pathways that emerge during clinical trials. It is important to search for new diagnostic methods and specific markers that could speed up the diagnosis of this group of patients, contribute to a quick accurate diagnosis, facilitate therapeutic decisions and could also be a prognostic factor for the further course of the disease. The search continues for methods of patient monitoring, which would be characterized by non-invasiveness and would allow for frequent follow-ups. Examinations should be extended to include the dynamic assessment of the cytokine networks during the disease’s course. It is also essential to observe how patients react to a specific therapy, which may make it easier to select patients for each treatment. Such a personalized medicine approach tailored individually to the patient’s needs seems to have the best chance of achieving the intended long-term goals and the expected clinical results [30].

### Limitations of the Study

The present study has some limitations. Firstly, the number of CD patients is relatively low; from this perspective, the results should be intended as exploratory in this specific cohort. Lastly, the results provide preliminary insight into the utility of using selected inflammatory markers in correlation with MRE for Crohn’s diseases diagnosis. A larger cohort would be necessary to confirm these findings and to assess the broader applicability of MRE across diverse patient subgroups. We are actively planning to increase the study group in future research to include a larger and more diverse population. This expansion could improve the generalizability of our findings and perhaps tailor a personalized treatment strategy.

## 5. Conclusions

The analysis of inflammatory marker concentrations and their correlations with radiological parameters in patients with CD indicates the following:

1. The determination of cytokine concentrations, such as IL-1β, IL-2, IL-4, IL-6, IL-7, IL-8, IL-9, IL-10, IL-13, IL-17, eotaxin, IFN-γ, IP-10, MCP-1, MIP-1β, PDGF-BB, RANTES, TNF-α VEGF and IL-1RA, can be a valuable tool in the diagnosis and assessment of CD activity. These markers can help diagnose the disease and monitor its course.

2. The concentrations of PDGF-BB, RANTES, IL-10 and IL-12 showed correlations with radiological parameters assessed by MRE, which characterize active inflammation. These cytokines have a particular potential as markers for the assessment of CD activity.

3. MRE parameter values showed no significant correlation with fecal calprotectin levels or disease severity as assessed by the CDAI. None of these indicators can be used as a single indicator of CD activity.

Research on the mechanisms of cytokine action in CD may lead to finding more effective treatment methods and contribute to faster diagnosis. The combined assessment of many inflammatory markers, including cytokines, with imaging results, may be more effective in assessing disease activity. This can lead to the development of more precise diagnostic tools that may potentially reduce the need for invasive examinations.

## Figures and Tables

**Table 1:** Clinical characteristics and the basic laboratory findings of the study group.

Variable	N	Min	Max	Median	Q1	Q3	Mean	SD	SE
Age (years)	24	22	78	43.5	31.5	57.8	45	15.8	3.23
BMI (kg/m^2^)	24	13.5	32	21.8	19.5	24.1	22.1	4.27	0.871
Height (m)	24	1.57	1.94	1.7	1.63	1.76	1.7	0.099	0.02
Body weight (kg/m^2^)	24	35	107	62	56.8	71.8	66	16.3	3.32
CDAI	24	88	408	214	165	274	226	92.5	18.9
WBC	24	3.65	25.1	7.04	4.96	9.66	8.06	5.02	1.02
Hematocrit	24	25.7	47.4	36	30.5	40.5	35.6	6.12	1.25
ESR—erythrocyte sedimentation rate	18	5	71	16	11.2	27.2	21.5	17.1	4.04
CRP	24	1	195	22.7	7.48	76.6	48.7	53.7	11

SD—standard deviation; SE—standard error; Q1—first quartile, Q3—third quartile.

**Table 2:** Calprotectin concentrations in the study group.

Variable	n	Min	Max	Median	Q1	Q3	Mean	SD	SE
Calprotectin (μg/g)	24	1.57	1162	299	65.9	878	442	418	85.4

**Table 3:** The presence of pANCA and cANCA antibodies in the study group.

pANCA	Negative	17	70.80%
	Positive	6	20.80%
	No data	2	8.33%
cANCA	Negative	22	91.70%
	Positive	0	0.00%
	No data	2	8.33%

**Table 4:** General characteristics of the control group.

Variable	n	Min	Max	Median	Q1	Q3	Mean	SD	SE
Age (years)	23	50	85	64	59.5	71.5	65.4	9.98	2.08
BMI (kg/m^2^)	23	20.1	44.1	24.3	23.4	27.6	26	4.85	1.01
Height (m)	23	1.52	1.83	1.67	1.62	1.72	1.67	0.077	0.016
Body weight (kg)	23	50	120	71	63.5	79	72.3	14.2	2.96
Hematocrit	23	29.9	45.8	41.8	40.1	43.7	41.4	3.48	0.726
WBC	23	3.56	10.8	7.32	5.42	8.3	6.94	1.84	0.383

SD—standard deviation; SE—standard error; Q1—first quartile, Q3—third quartile.

**Table 5:** Comparison of the basic parameters of the study and control groups.

Variable	Study Group (n = 24)			Control Group (n = 23)			*p*
	Median	Q1	Q3	Median	Q1	Q3	
Age (years)	43.50	31.00	58.50	64.00	59.00	72.00	0.00
Height (m)	1.70	1.63	1.77	1.67.	1.61	1.72	0.42
Body weight (kg)	62.00	56.50	72.50	71.00	63.00	79.00	0.08
BMI (kg/m^2^)	21.85	19.42	24.20	24.30	23.26	27.89	0.00
WBC	7.04	4.84	9.67	7.32	5.38	8.36	0.99
Hematocrit	36.05	30.35	40.50	41.80	39.70	43.70	0.00

**Table 6:** Comparison of interleukin concentrations in the study Group (S) and the Group (C).

Variable	Group S			Group C			*p*
	Median	Q1	Q3	Median	Q1	Q3	
IL-1β	5.19	3.84	7.55	2.18	1.06	3.84	0.00
IL-1RA	284.59	180.45	583.97	123.75	36.21	162.93	0.00
IL-2	4.52	1.96	6.19	0.62	0.56	0.68	0.00
IL-4	4.12	3.28	5.16	2.92	2.44	3.35	0.00
IL-5	6.86	4.92	14.70	5.61	4.98	6.86	0.26
IL-6	1.48	0.82	3.75	0.18	0.18	0.20	0.00
IL-7	124.25	92.72	181.64	72.30	46.62	151.49	0.01
IL-8	25.00	14.53	46.35	2.82	0.07	20.46	0.00
IL-9	254.19	161.49	286.77	109.76	63.56	147.18	0.00
IL-10	4.28	2.96	6.34	1.01	0.02	4.42	0.00
IL-12 (p70)	1.71	0.36	2.20	1.71	1.71	1.95	0.52
IL-13	11.47	7.97	23.99	8.40	4.60	10.89	0.03
IL-15	198.06	110.02	283.01	123.78	110.02	130.65	0.08
IL-17A	5.54	3.96	6.90	0.55	0.55	2.92	0.00

Cytokine concentrations are expressed in pg/mL.

**Table 7:** Comparison of the concentrations of other cytokines in Group S and Group C.

Variable	Group S			Group C			*p*
	Median	Q1	Q3	Median	Q1	Q3	
Eotaxin	95.94	61.92	123.45	53.49	19.03	101.17	0.02
FGF-Basic	65.08	50.87	81.77	51.64	34.32	76.54	0.29
G-CSF	75.52	50.31	140.06	76.56	55.85	124.26	0.95
GM-CSF	2.49	0.50	4.01	1.87	1.66	1.97	0.45
IFN-γ	2.35	1.51	5.00	0.49	0.45	1.75	0.01
IP-10	362.55	226.93	449.42	49.38	26.27	278.70	0.00
MCP-1 (MCAF)	30.98	22.12	49.05	1.67	1.51	11.25	0.00
MIP-1α	5.35	4.08	8.78	4.68	2.75	6.82	0.24
MIP-1β	90.65	81.13	120.84	33.82	17.09	48.03	0.00
PDGF-BB	3580.42	2190.98	6371.61	292.35	65.40	1226.16	0.00
RANTES	2615.87	1662.23	3657.45	855.64	515.59	1780.38	0.00
TNF α	8.88	4.82	12.09	0.35	0.31	0.35	0.00
VEGF	64.97	28.26	152.46	17.53	15.34	21.92	0.00

Cytokine concentrations are expressed in pg/mL. *p*—*p*-value (test probability), Q1—first quartile, Q3—third quartile.

## Data Availability

The original contributions presented in this study are included in the article/Supplementary Material. Further inquiries can be directed to the corresponding author(s).

## Supplementary Materials

The following supporting information can be downloaded at: https://www.mdpi.com/article/10.3390/biom15010116/s1.
