# Supplementary material for: Assessment of the Utility of Selected Inflammatory Markers in Correlation with Magnetic Resonance Enterography (MRE) Findings in the Diagnosis of Crohn’s Disease
Source: Biomolecules. 2025 Jan 13;15(1):116. doi: 10.3390/biom15010116 (PMC11763748; doi:10.3390/biom15010116)

The principal components method with Varimax rotation was used for PCA analysis. Two principal components were obtained and used as variables in further analysis.

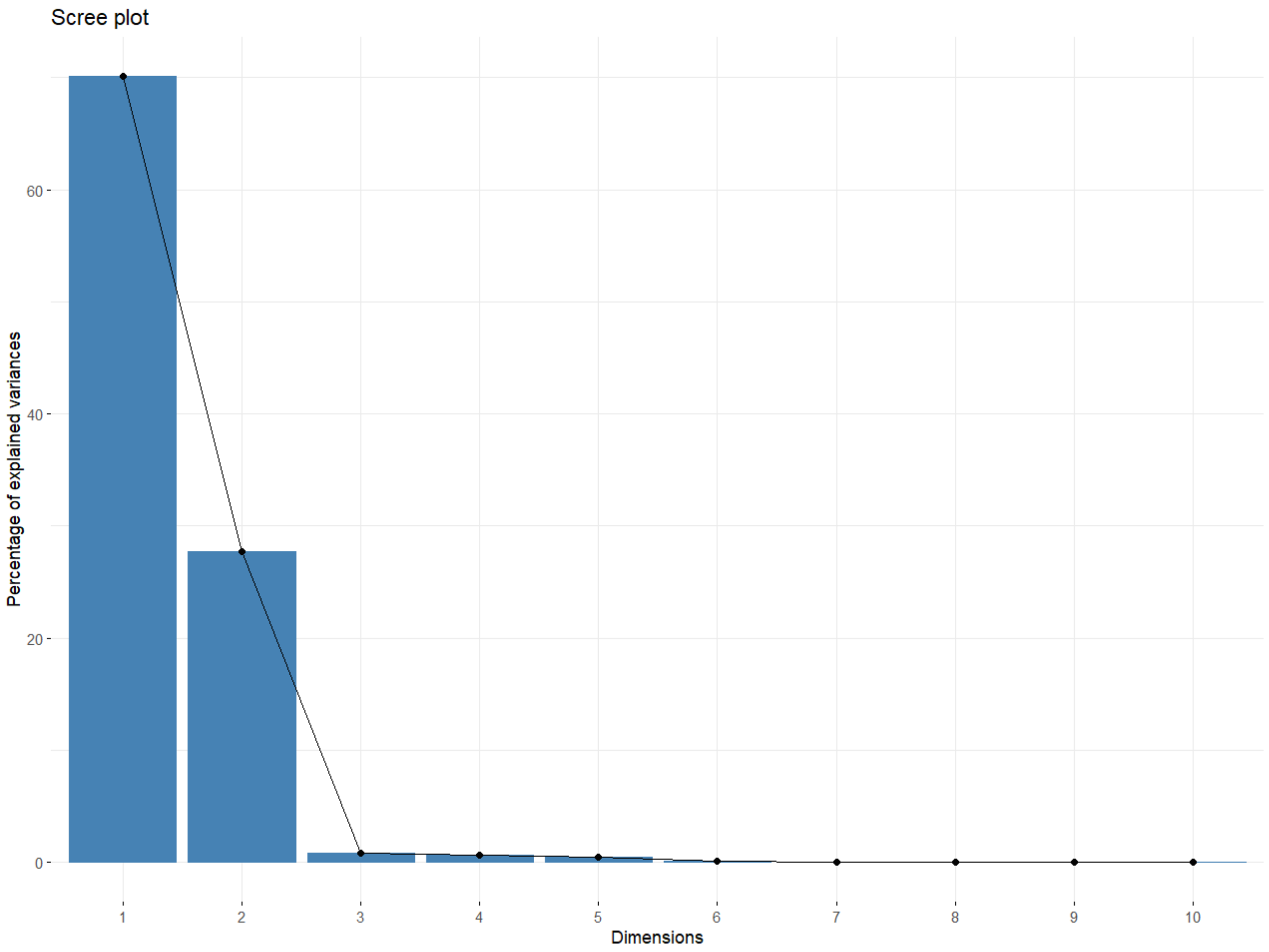

Supplement: Supplementary file 1 [file biomolecules-15-00116-s001.zip › PCA.pdf]
