# Supplementary material for: Assessment of the Utility of Selected Inflammatory Markers in Correlation with Magnetic Resonance Enterography (MRE) Findings in the Diagnosis of Crohn’s Disease
Source: Biomolecules. 2025 Jan 13;15(1):116. doi: 10.3390/biom15010116 (PMC11763748; doi:10.3390/biom15010116)
Supplement: Supplementary file 1 [file biomolecules-15-00116-s001.zip › power analysis.pdf]

The table shows the results of the power analysis. test. The first column indicates the effect size (up to 0.3 weak, 0.31-0.6 medium, above 0.6 strong) and the resulting power analyses. In most variables, the power is quite high. Power of 0.8 is considered desirable and has been achieved in many cytokines.

Variable	effect size	power
IL -1b	0,5081207	0,66418531
IL -1ra	0,61351438	0,82076759
IL-2	0,68114291	0,89168241
IL-4	0,55594399	0,7415855
IL-5	0,1410971	0,10152164
IL-6	0,50138314	0,6525851
IL-7	0,3977786	0,46272334
IL-8	0,479	0,61306621
IL-9	0,687	0,89670524
IL-10	0,47910362	0,61325226
IL-12 (p70)	0,17151065	0,12683928
IL-13	0,3111252	0,30875962
IL-15	0,2358368	0,19778113
IL -17A	0,65882055	0,8709338
Eotaxin	0,34524736	0,36703272
FGF Basic	0,15053538	0,10881455
G-CSF	0,015232	0,05058527
GM-CSF	0,05108391	0,05660645

IFN-g	0,42080889	0,50574709
IP-10	0,56198423	0,75066214
MCP-1 (MCAF)	0,68643575	0,89622885
MIP-1a	0,20997384	0,16644067
MIP-1b	0,823	0,9713439
PDGF-bb	0,73452102	0,93134791
RANTES	0,6464926	0,8583619
TNF-a	0,76013703	0,94586968
VEGF	0,44275016	0,54668917

Test power analysis was performed using the PWR package for R in the RStudio environment.
