# Supplementary material for: Assessment of the Utility of Selected Inflammatory Markers in Correlation with Magnetic Resonance Enterography (MRE) Findings in the Diagnosis of Crohn’s Disease
Source: Biomolecules. 2025 Jan 13;15(1):116. doi: 10.3390/biom15010116 (PMC11763748; doi:10.3390/biom15010116)
Supplement: Supplementary file 1 [file biomolecules-15-00116-s001.zip › tables_PCA.pdf]

Principal Component Analysis Results for variables:
1 "coord" - coordinates for the variables
2 "contrib" -contributions of the variables
3 "cos2" - cos2 for the variables

Coord

| Dim.1 | Dim.2 | Dim.3 | Dim.4 | Dim.5 | Dim.6 | Dim.7 | Dim.8 | Dim.9 | Dim.10 | Dim.11 | Dim.12 | Dim.13 | Dim.14 | Dim.15 | Dim.16 | Dim.17 | Dim.18 | Dim.19 | Dim.20 | Dim.21 | Dim.22 | Dim.23 | Dim.24 | Dim.25 | Dim.26 | Dim.27 |
|---|---|---|---|---|---|---|---|---|---|---|---|---|---|---|---|---|---|---|---|---|---|---|---|---|---|---|
| -0,7994707 | 0,16906028 | -4,5642070 | -1,7729210 | -1,7649750 | 5,22793879 | -0,0220289 | 4,07164808 | -0,4634139 | 0,79774896 | -0,7560169 | 0,88306384 | 0,36190096 | 0,4648999 | -0,1656924 | -0,3760278 | -0,1526750 | -0,8367100 | 0,34832238 | 0,70743743 | 0,95272196 | 0,24089085 | 0,37713784 | 0,44937138 | 0,29161279 | 0,13245184 | 0,04178456 |
| -205,27807 | -86,741002 | -64,388901 | -41,717362 | -234,87331 | -33,743207 | -0,6466597 | 2,17668638 | -2,0849524 | 0,06815228 | 1,17803651 | -0,9013426 | -1,0254966 | 0,24076453 | 0,04852482 | -0,2064976 | -0,0536838 | 0,01256878 | -0,0050112 | 0,00624367 | -0,0143645 | 0,00330830 | 0,00376550 | 0,00586273 | 0,00220490 | -0,0023204 | -0,0028529 |
| -0,7152620 | -0,0912260 | -3,3535568 | -0,6030143 | -1,3199283 | 4,20339037 | -0,4377697 | 1,78781328 | -0,7323878 | 0,54880994 | -0,1353975 | 0,36822540 | -0,0039276 | -0,3578194 | -0,0951173 | -0,5403926 | 0,36395570 | -0,1702374 | 0,43627193 | 0,35848285 | 0,33350849 | 0,00997428 | -0,5957000 | -0,2808903 | 0,52986060 | -0,1869199 | -0,1077417 |
| -0,4691608 | 0,21710301 | -1,3409407 | -0,2420822 | -0,3500597 | 1,02497321 | -0,2256027 | 0,65795925 | -0,0772698 | 0,12839498 | 0,02927181 | 0,00659292 | -0,1529612 | -0,1414292 | -0,3113461 | -0,1425224 | -0,1595404 | 0,11607414 | 0,07493353 | 0,03770595 | -0,0312801 | -0,0109346 | 0,23350475 | -0,0750669 | 0,09952719 | -0,3834134 | 0,31459645 |
| -0,4367809 | 2,39994754 | -19,223223 | -2,8804207 | -8,0911152 | 22,9415597 | -1,2388536 | 12,7283534 | 0,59302961 | -3,6334114 | -2,8426076 | 4,85300396 | -0,9110202 | -6,8024979 | -3,3528502 | -4,6113606 | -1,3276275 | -0,7016418 | -0,2824030 | -0,2492808 | -0,3296669 | 0,00901322 | 0,08901911 | 0,05355668 | 0,06757565 | -0,0128447 | -0,0387161 |
| -1,3022954 | -0,3614908 | -3,8431405 | 2,49630455 | -5,7335390 | -2,1189405 | 2,14893764 | 3,23986374 | 1,77893095 | -2,0930661 | 12,0309587 | 3,83640411 | 16,8422298 | -0,9803259 | 0,75514900 | -0,3897056 | 0,01232505 | -0,2084074 | -0,0225317 | -0,1167570 | 0,02136969 | 0,02043674 | -0,0350054 | 0,00060327 | -0,0071910 | 0,00389572 | 0,01307940 |
| -33,066366 | 42,8822619 | -252,79500 | -134,10352 | 63,9477889 | 3,12979049 | 3,66735712 | 11,4712421 | -9,1035312 | -2,0487098 | 3,26560130 | -3,5825110 | -1,2058455 | 1,31590569 | 1,10980280 | -0,2325043 | -0,3302729 | -0,1551959 | -0,0750356 | 0,09807464 | -0,0863231 | 0,00318215 | -0,0132434 | 0,00135820 | -0,0167545 | -0,0045469 | -0,0016726 |
| -11,896644 | -3,8198568 | -9,1758045 | 5,87769184 | -4,3673387 | 1,95996944 | -7,8530063 | 4,36213531 | -0,6346860 | -3,6410876 | -0,8877937 | 8,41668447 | -2,9371160 | -6,6646660 | 5,77947363 | 2,71752690 | -1,4825574 | 0,34183584 | 0,08022439 | -0,1031378 | 0,14115043 | 0,01628991 | 0,01636715 | -0,0151096 | 0,01350952 | -0,0091173 | -0,0031781 |
| -63,390655 | 10,5857167 | -40,520798 | 22,4805415 | 3,05336306 | 8,15951284 | -60,995416 | -11,618501 | -6,0574997 | 2,37203346 | 3,08642455 | 2,51929051 | -0,2639180 | 1,34785798 | -1,2798466 | 0,07725025 | -0,1609712 | -0,1788771 | -0,0258698 | 0,00098513 | -0,0389564 | 0,00293598 | -0,0110026 | 0,01737742 | -0,0005403 | 0,00139345 | -0,0002745 |
| -0,4803389 | 3,16001833 | -12,016840 | -6,5063320 | 2,21191261 | 0,68083964 | -0,4324132 | 0,94494679 | 0,73519232 | -0,2630695 | 0,39248320 | -0,6243449 | -0,6675153 | 1,19836939 | -0,0973602 | -0,2875870 | 0,05931090 | -0,5821985 | 0,42233702 | -1,4794905 | 0,81305845 | -0,4968474 | 0,22995277 | -0,0608646 | -0,0432030 | -0,1080043 | -0,0860795 |
| 1,54251300 | 2,45562000 | -13,535741 | -5,7722431 | 1,04054509 | 5,85973513 | -3,9332461 | 1,99606052 | -1,8961815 | -0,3840511 | 2,60417873 | 1,52575398 | -2,3340067 | -4,2425916 | 1,21527480 | -1,7501630 | 4,99555303 | 0,57826797 | 0,14671769 | 0,00087051 | 0,02796813 | -0,0237347 | 0,09666622 | 0,01133653 | -0,0195527 | 0,02838360 | 0,01747198 |
| -3,0743698 | 2,53565304 | -19,521023 | -8,8472406 | 4,60562390 | 0,90533453 | -0,3877424 | -0,8442280 | -1,0645602 | 0,42564777 | 0,28037369 | 0,07621149 | 1,29706509 | -0,5489282 | -1,5975563 | -1,3006304 | -1,1489224 | 3,43258124 | 0,63765810 | 0,01577477 | 0,21708584 | -0,1341972 | 0,03310333 | 0,10060081 | 0,03928635 | 0,04208492 | -0,0041621 |
| -46,628143 | -20,373211 | -82,246086 | 26,6877067 | -46,354259 | 94,3056729 | 12,9267831 | -25,728090 | 5,31166571 | 8,23586258 | 2,98321403 | -0,2778501 | -0,7175254 | -0,7201496 | 0,64825681 | 0,03199512 | -0,2508228 | -0,0542890 | -0,0450618 | 0,00196191 | -0,0003507 | -0,0075264 | 0,01259417 | -0,0030151 | 0,00005503 | 0,00227450 | -0,0003281 |
| -1,5250321 | 0,40811861 | -4,2279653 | -1,7471912 | -2,3785532 | 4,26027851 | -0,9589425 | 2,28574042 | -0,2862865 | -0,0772850 | -0,1166462 | 0,60782404 | 0,14395964 | -0,5517660 | -0,7671735 | -0,6931583 | -0,3620754 | -0,2151724 | 0,71657247 | 0,47176749 | 0,38903227 | 0,40697123 | 0,27906712 | -0,7248145 | -0,2712861 | 0,05682374 | -0,0137701 |
| -14,508873 | 19,1582252 | -18,407860 | 3,66373409 | 0,40158431 | 3,83385774 | -15,840240 | 18,1190044 | 29,7932110 | 1,35324355 | 3,61412000 | -5,8736692 | -2,2261201 | 1,14198942 | 1,94595994 | -1,4072676 | -0,2610768 | 0,14418702 | 0,02798452 | 0,09527042 | -0,0327251 | 0,00002545 | -0,0227322 | 0,00613483 | -0,0087356 | 0,00378887 | 0,00394485 |
| -1,8396252 | 1,70559120 | -24,746472 | -6,8287734 | -15,229005 | 29,1922182 | 2,20145867 | 28,2967040 | 2,80835007 | 9,13186544 | -2,1391198 | 12,0608101 | -0,0704031 | 3,58100427 | -2,3730659 | 2,92592194 | 0,82046504 | 0,29312660 | -0,0678630 | -0,1350004 | -0,0841287 | 0,03006747 | -0,0111952 | -0,0179999 | -0,0041814 | 0,00379464 | -0,0006652 |
| -15,986122 | 20,7503549 | -114,65935 | -50,881539 | 30,7253353 | -54,425984 | 3,06036188 | -24,716195 | 16,5788451 | 2,40566981 | -6,4866773 | 6,56535395 | 2,21600040 | -2,1773004 | -1,9567766 | 0,95105687 | 0,48392159 | -0,1404580 | -0,0031012 | -0,0676604 | 0,06032782 | 0,05431288 | -0,0060233 | -0,0122248 | 0,03226650 | 0,01008334 | 0,00851690 |
| -0,4392561 | -0,1165880 | -2,8400525 | -0,0666691 | -1,5589373 | 3,25120513 | 0,14368852 | 0,85924745 | 0,45165863 | -0,3907427 | -0,4182985 | 0,73441819 | 0,05503779 | -0,8955762 | -0,5826413 | -0,2113998 | 0,12208444 | -0,0124044 | 0,09439750 | 0,43517768 | 0,42720917 | 0,27350993 | -0,2705370 | 0,37017613 | -0,5017698 | -0,3047682 | -0,1096337 |
| -0,6595817 | -0,0004288 | -4,4563995 | -1,5334591 | -3,8621250 | 4,77396588 | -0,0607897 | 3,13684353 | -0,3460990 | -0,5736208 | -0,1843652 | 0,87739048 | -1,0534720 | -1,4865414 | -0,9435581 | -0,5611174 | -0,2108304 | -0,2828268 | -0,3628633 | 0,19467860 | 0,68126015 | -0,4427331 | -0,5628502 | -0,0846714 | -0,2173150 | 0,19534171 | 0,20364950 |
| -123,17777 | 55,2003338 | -147,06931 | 266,751433 | 6,45577032 | -24,085634 | 6,95439052 | 5,17214291 | -2,1472721 | -1,2558158 | 0,43990608 | -0,2322097 | -0,6927411 | 0,43448263 | -0,0551251 | -0,1759730 | 0,04332957 | 0,01117287 | -0,0018086 | 0,00929267 | 0,00181018 | -0,0003644 | 0,00176498 | -0,0020027 | -0,0013428 | -0,0003045 | -0,0007810 |
| -12,905653 | 2,72701117 | -7,7540224 | 4,86228999 | -1,8075212 | 5,83081305 | -3,8121511 | 7,56786468 | 1,04990758 | 1,07523987 | 3,05702700 | -10,626924 | 0,95569898 | -6,9677927 | -3,6455358 | 4,79860121 | 0,18385548 | 0,00186125 | -0,0654371 | 0,02131137 | 0,06812208 | -0,0352976 | 0,07265345 | -0,0100077 | 0,04751487 | -0,0055759 | -0,0212276 |
| -2,2492723 | -0,2278160 | -1,3491482 | 0,46558370 | -1,8208317 | -0,7618696 | 0,03166169 | 0,21335804 | 0,85033378 | 0,33752914 | -1,1637938 | 1,96844957 | 1,34922551 | -0,1257254 | 0,14912377 | 0,22072333 | -0,0400056 | -0,5088421 | 0,55471963 | 1,16859235 | -0,3234181 | -0,9083918 | 0,23466970 | -0,0078488 | -0,0746450 | -0,0585754 | -0,0714413 |
| -29,958674 | 1,88036648 | -8,7796304 | 5,51578028 | 1,66897317 | -7,8170718 | -6,7859414 | 7,48719261 | -5,4850651 | 19,0213995 | -11,674545 | -5,6294493 | 6,91115601 | -2,0856045 | 2,33161639 | -1,3242391 | -0,0526361 | -0,0313582 | -0,0608267 | -0,1035781 | 0,00969233 | -0,0030769 | -0,0165621 | -0,0102393 | -0,0357382 | -0,0010095 | 0,00622550 |
| -2705,5797 | -1000,3711 | 10,0038120 | -3,2604413 | 19,6537434 | 0,81157317 | 0,76735190 | 0,25671760 | 0,23158689 | -0,1024547 | 0,04911351 | 0,10594761 | -0,0238109 | 0,00593365 | -0,0294085 | -0,0128071 | 0,01170304 | -0,0010381 | -0,0001190 | -0,0008156 | 0,00087473 | -0,0001600 | 0,00002377 | -0,0002729 | 0,00001730 | 0,00005470 | 0,00004777 |
| -1592,2478 | 1705,82420 | 14,4929305 | -8,4535396 | -3,3384677 | 1,31233442 | 0,72929600 | -0,3815331 | -0,0888723 | -0,0510992 | -0,0172587 | 0,08987447 | 0,00487780 | -0,0165106 | 0,00306055 | 0,01117367 | 0,00237228 | 0,00199217 | -0,0005347 | -0,0002815 | 0,00063403 | 0,00028023 | 0,00004080 | 0,00002307 | 0,00027315 | 0,00000540 | 0,00000213 |
| -4,1895680 | -0,0952340 | -4,8951360 | 0,93758708 | -2,5378555 | 4,57505127 | 0,42645788 | 1,09769709 | -0,1678663 | -0,4187621 | -0,7572399 | -0,7477552 | -0,1769002 | -0,5746368 | -0,2251591 | 0,40779861 | -0,0496492 | -0,8243104 | 1,88582472 | -0,4368074 | -0,5100950 | 0,12687021 | -0,2675595 | 0,21321725 | -0,0646745 | 0,08867519 | 0,08904435 |
| -33,968451 | -9,0833396 | -39,140183 | 7,27708648 | -29,699516 | 42,6577740 | -8,9155418 | 2,11209471 | 2,89171335 | -17,062286 | -13,912517 | -2,6333948 | 6,60198089 | 1,73493264 | 0,35028084 | 0,92726150 | 0,79756580 | 0,24129831 | -0,0684569 | -0,0270278 | 0,03698294 | 0,00866999 | -0,0042627 | -0,0233336 | 0,00409910 | 0,00452080 | 0,00794200 |

Contrib

| Dim.1 | Dim.2 | Dim.3 | Dim.4 | Dim.5 | Dim.6 | Dim.7 | Dim.8 | Dim.9 | Dim.10 | Dim.11 | Dim.12 | Dim.13 | Dim.14 | Dim.15 | Dim.16 | Dim.17 | Dim.18 | Dim.19 | Dim.20 | Dim.21 | Dim.22 | Dim.23 | Dim.24 | Dim.25 | Dim.26 | Dim.27 |
|---|---|---|---|---|---|---|---|---|---|---|---|---|---|---|---|---|---|---|---|---|---|---|---|---|---|---|
| 0,00000644 | 0,00000072 | 0,01804464 | 0,00330349 | 0,00485992 | 0,15960619 | 0,00001092 | 0,54314463 | 0,01558624 | 0,07423189 | 0,09639200 | 0,15182174 | 0,03198050 | 0,11111695 | 0,03167652 | 0,19538463 | 0,07179593 | 4,61768908 | 2,18412909 | 10,0783975 | 29,7818992 | 3,61001120 | 11,6051591 | 19,7934290 | 11,0208143 | 4,87623890 | 0,92326818 |
| 0,42466577 | 0,19172102 | 3,59120072 | 1,82906244 | 86,0635820 | 6,64907871 | 0,00941590 | 0,15522681 | 0,31549717 | 0,00054177 | 0,23404302 | 0,15817199 | 0,25678772 | 0,02980200 | 0,00271681 | 0,05892243 | 0,00887668 | 0,00104198 | 0,00045207 | 0,00078504 | 0,00677022 | 0,00068089 | 0,00115694 | 0,00336907 | 0,00063005 | 0,00149658 | 0,00430402 |
| 0,00000515 | 0,00000021 | 0,00974157 | 0,00038210 | 0,00271802 | 0,10317820 | 0,00431521 | 0,10471751 | 0,03893008 | 0,03513193 | 0,00309171 | 0,02639838 | 0,00000370 | 0,06582470 | 0,01043883 | 0,40352384 | 0,40800060 | 0,19115464 | 3,42633711 | 2,58792800 | 3,64950394 | 0,00618915 | 28,9538372 | 7,73364122 | 36,3851184 | 9,71136310 | 6,13852493 |
| 0,00000221 | 0,00000120 | 0,00155753 | 0,00006159 | 0,00019117 | 0,00613498 | 0,00114603 | 0,01418310 | 0,00043333 | 0,00192288 | 0,00014450 | 0,00000840 | 0,00571305 | 0,01028347 | 0,11184562 | 0,02806835 | 0,07839808 | 0,08886805 | 0,10108062 | 0,02863092 | 0,03210388 | 0,00743830 | 4,44879413 | 0,55234193 | 1,28376054 | 40,8604940 | 52,3363918 |
| 0,00000192 | 0,00014670 | 0,32008852 | 0,00871980 | 0,10213364 | 3,07351004 | 0,03455815 | 5,30786022 | 0,02552442 | 1,53988124 | 1,36273699 | 4,58533499 | 0,20265713 | 23,7902071 | 12,9706072 | 29,3838345 | 5,42895565 | 3,24717492 | 1,43566863 | 1,25139118 | 3,56591435 | 0,00505392 | 0,64657281 | 0,28114970 | 0,59180840 | 0,04585858 | 0,79264899 |
| 0,00001709 | 0,00000332 | 0,01279352 | 0,00654922 | 0,05128585 | 0,02621961 | 0,10398201 | 0,34389710 | 0,22967902 | 0,51100362 | 24,4106123 | 2,86548720 | 69,2635861 | 0,49408570 | 0,65795667 | 0,20985708 | 0,00046788 | 0,28648455 | 0,00913915 | 0,27452467 | 0,01498362 | 0,02598313 | 0,09998224 | 0,00003567 | 0,00670168 | 0,00421838 | 0,09046328 |
| 0,01101882 | 0,04685718 | 55,3547170 | 18,9005850 | 6,37974225 | 0,05720300 | 0,30284261 | 4,31117760 | 6,01482407 | 0,48957470 | 1,79847554 | 2,49876182 | 0,35504971 | 0,89024781 | 1,42109660 | 0,07469860 | 0,33597714 | 0,15886774 | 0,10135623 | 0,19369969 | 0,24449771 | 0,00062995 | 0,01431042 | 0,00018083 | 0,03638047 | 0,00574647 | 0,00147940 |
| 0,00142630 | 0,00037180 | 0,07292995 | 0,03630851 | 0,02975677 | 0,02243300 | 1,38861704 | 0,62340932 | 0,02923620 | 1,54639465 | 0,13292369 | 13,7921467 | 2,10643470 | 22,8359010 | 38,5397202 | 10,2046471 | 6,76997243 | 0,77074354 | 0,11585864 | 0,21421590 | 0,65370905 | 0,01650841 | 0,02185734 | 0,02237801 | 0,02365269 | 0,02310482 | 0,00534118 |
| 0,04049604 | 0,00285530 | 1,42224247 | 0,53113924 | 0,01454484 | 0,38879190 | 83,7731791 | 4,42257533 | 2,66311531 | 0,65629590 | 1,60653254 | 1,23568074 | 0,01700763 | 0,93400598 | 1,88993920 | 0,00824613 | 0,07981060 | 0,21104958 | 0,01204769 | 0,00001954 | 0,04979430 | 0,00053625 | 0,00987750 | 0,02959927 | 0,00003783 | 0,00053970 | 0,00003985 |
| 0,00000232 | 0,00025444 | 0,12508297 | 0,04449050 | 0,00763287 | 0,00270693 | 0,00421025 | 0,02925430 | 0,03922880 | 0,00807233 | 0,02597888 | 0,07589252 | 0,10879985 | 0,73831688 | 0,01093692 | 0,11428488 | 0,01083509 | 2,23571793 | 3,21095230 | 44,0798219 | 21,6902008 | 15,3572680 | 4,31447712 | 0,36311205 | 0,24189678 | 3,24228412 | 3,91828734 |
| 0,00002397 | 0,00015365 | 0,15870175 | 0,03501741 | 0,00168917 | 0,20051401 | 0,34834760 | 0,13053363 | 0,26095342 | 0,01720427 | 1,14372015 | 0,45323010 | 1,33017875 | 9,25387971 | 1,70404483 | 4,23260059 | 76,8654117 | 2,20563168 | 0,38750784 | 0,00001520 | 0,02566533 | 0,03504602 | 0,76243071 | 0,01259710 | 0,04954678 | 0,22392567 | 0,16142868 |
| 0,00009525 | 0,00016383 | 0,33008274 | 0,08226413 | 0,03309245 | 0,00478637 | 0,00338530 | 0,02335041 | 0,08225140 | 0,02113288 | 0,01325722 | 0,00113081 | 0,41078990 | 0,15491464 | 2,94472223 | 2,33753492 | 4,06579244 | 77,7170587 | 7,31966670 | 0,00501120 | 1,54626188 | 1,12035332 | 0,08941171 | 0,99200353 | 0,20002492 | 0,49229125 | 0,00916057 |
| 0,02191080 | 0,01057644 | 5,85933240 | 0,74854405 | 3,35221109 | 51,9355088 | 3,76262999 | 21,6865327 | 2,04769197 | 7,91181741 | 1,50088364 | 0,01503041 | 0,12571307 | 0,26662860 | 0,48487094 | 0,00141454 | 0,19377511 | 0,01944014 | 0,03655390 | 0,00007751 | 0,00000403 | 0,00352408 | 0,01294160 | 0,00089111 | 0,00000039 | 0,00143794 | 0,00005695 |
| 0,00002343 | 0,00000424 | 0,01548390 | 0,00320830 | 0,00882628 | 0,10598990 | 0,02070597 | 0,17117040 | 0,00594840 | 0,00069670 | 0,00229460 | 0,07192925 | 0,00506042 | 0,15652052 | 0,67907740 | 0,66391913 | 0,40379603 | 0,30538535 | 9,24348560 | 4,48199572 | 4,96582370 | 10,3037517 | 6,35430738 | 51,4948733 | 9,53796379 | 0,89748798 | 0,10027062 |
| 0,00212143 | 0,00935257 | 0,29351091 | 0,01410720 | 0,00025159 | 0,08583427 | 5,64981380 | 10,7558240 | 64,4225307 | 0,21360430 | 2,20284207 | 6,71689800 | 1,21004911 | 0,67047957 | 4,36917839 | 2,73655187 | 0,20994251 | 0,13712828 | 0,01409779 | 0,18278125 | 0,03513840 | 0,00000004 | 0,04216348 | 0,00368900 | 0,00988985 | 0,00399015 | 0,00822921 |
| 0,00003410 | 0,00007412 | 0,53044975 | 0,04900949 | 0,36182217 | 4,97648787 | 0,10912680 | 26,2329250 | 0,57240793 | 9,72695887 | 0,77169987 | 28,3206180 | 0,00121029 | 6,59281760 | 6,49757963 | 11,8297552 | 2,07340420 | 0,56674170 | 0,08290521 | 0,36701697 | 0,23222503 | 0,05624213 | 0,01022631 | 0,03175799 | 0,00226590 | 0,00400231 | 0,00023403 |
| 0,00257542 | 0,01097164 | 11,3877154 | 2,72091731 | 1,47280527 | 17,2982168 | 0,21089008 | 20,0142013 | 19,9485670 | 0,67504093 | 7,09615001 | 8,39201145 | 1,19907262 | 2,43723899 | 4,41788589 | 1,24986545 | 0,72129647 | 0,13012709 | 0,00017313 | 0,09219038 | 0,11941403 | 0,18351580 | 0,00296024 | 0,01464852 | 0,13492923 | 0,02826038 | 0,03835882 |
| 0,00000194 | 0,00000034 | 0,00698667 | 0,00000467 | 0,00379148 | 0,06172733 | 0,00046489 | 0,02418864 | 0,01480552 | 0,01780901 | 0,02950870 | 0,10501144 | 0,00073965 | 0,41235053 | 0,39168281 | 0,06175323 | 0,04590758 | 0,00101492 | 0,16041210 | 3,81371870 | 5,98826718 | 4,65386885 | 5,97178870 | 13,4315788 | 32,6294421 | 25,8171599 | 6,35601404 |
| 0,00000438 | 0,00000000 | 0,01720227 | 0,00247137 | 0,02327044 | 0,13309059 | 0,00008320 | 0,32237454 | 0,00869368 | 0,03838025 | 0,00573240 | 0,14987721 | 0,27098910 | 1,13609702 | 1,02723382 | 0,43506873 | 0,13690858 | 0,52761345 | 2,37029155 | 0,76322418 | 15,2281103 | 12,1941624 | 25,8485770 | 0,70272310 | 6,12040602 | 10,6061782 | 21,9312359 |
| 0,15290682 | 0,07764334 | 18,7353497 | 74,7839458 | 0,06502018 | 3,38769997 | 1,08900215 | 0,87642762 | 0,33463959 | 0,18395431 | 0,03263609 | 0,01049809 | 0,11717844 | 0,09705238 | 0,00350610 | 0,04279004 | 0,00578272 | 0,00082338 | 0,00005889 | 0,00173898 | 0,00010757 | 0,00000820 | 0,00025417 | 0,00039310 | 0,00023369 | 0,00002577 | 0,00032250 |
| 0,00167850 | 0,00018949 | 0,05208009 | 0,02484714 | 0,00509704 | 0,19853952 | 0,32722820 | 1,87638407 | 0,08000282 | 0,13485554 | 1,57607458 | 21,9869488 | 0,22302217 | 24,9604160 | 15,3339740 | 31,8184928 | 0,10411588 | 0,00002284 | 0,07708390 | 0,00914614 | 0,15226353 | 0,07751020 | 0,43068857 | 0,00981712 | 0,29259019 | 0,00864195 | 0,23828723 |
| 0,00005098 | 0,00000132 | 0,00157665 | 0,00022781 | 0,00517239 | 0,00338961 | 0,00002257 | 0,00149139 | 0,05247857 | 0,01328864 | 0,22841798 | 0,75439424 | 0,44450319 | 0,00812658 | 0,02565817 | 0,06732041 | 0,00492953 | 1,70781533 | 5,53939857 | 27,5005579 | 3,43201167 | 51,3350925 | 4,49329721 | 0,00603842 | 0,72210785 | 0,95367558 | 2,69895508 |
| 0,00904497 | 0,00009009 | 0,06676820 | 0,03197480 | 0,00434561 | 0,35684281 | 1,03688551 | 1,83659340 | 2,18356805 | 42,2029692 | 22,9857229 | 6,16994941 | 11,6629170 | 2,23627562 | 6,27257960 | 2,42316571 | 0,00853359 | 0,00648601 | 0,06660471 | 0,21604859 | 0,00308231 | 0,00058898 | 0,02238129 | 0,01027660 | 0,16552629 | 0,00028330 | 0,02049480 |
| 73,7705160 | 25,5001737 | 0,08668594 | 0,01117242 | 0,60261874 | 0,00384630 | 0,01325860 | 0,00215910 | 0,00389252 | 0,00122439 | 0,00040679 | 0,00218540 | 0,00013843 | 0,00001810 | 0,00099788 | 0,00022665 | 0,00042185 | 0,00000710 | 0,00000025 | 0,00001339 | 0,00002510 | 0,00000159 | 0,00000004 | 0,00000730 | 0,00000003 | 0,00000083 | 0,00000120 |
| 25,5495640 | 74,1462904 | 0,18194078 | 0,07510555 | 0,01738787 | 0,01005721 | 0,01197617 | 0,00476913 | 0,00057323 | 0,00030457 | 0,00005023 | 0,00157261 | 0,00000580 | 0,00014014 | 0,00001080 | 0,00017252 | 0,00001733 | 0,00002617 | 0,00000514 | 0,00000159 | 0,00001319 | 0,00000488 | 0,00000013 | 0,00000005 | 0,00000960 | 0,00000000 | 0,00000000 |
| 0,00017688 | 0,00000023 | 0,02075610 | 0,00092388 | 0,01004814 | 0,12223080 | 0,00409508 | 0,03947662 | 0,00204517 | 0,02045468 | 0,09670417 | 0,10886009 | 0,00764121 | 0,16976508 | 0,05849400 | 0,22979563 | 0,00759258 | 4,48183962 | 64,0203702 | 3,84233713 | 8,53733239 | 1,00135321 | 5,84106372 | 4,45610003 | 0,54208420 | 2,18561297 | 4,19284591 |
| 0,01162823 | 0,00210238 | 1,32697689 | 0,05565573 | 1,37610265 | 10,6263749 | 1,78980648 | 0,14615100 | 0,60689511 | 33,9572529 | 32,6429670 | 1,35014915 | 10,6427612 | 1,54748562 | 0,14156788 | 1,18810479 | 1,95928158 | 0,38404602 | 0,08436270 | 0,01471080 | 0,04487691 | 0,00467633 | 0,00148263 | 0,05336712 | 0,00217767 | 0,00568082 | 0,03335512 |

Cos2

| Dim.1 | Dim.2 | Dim.3 | Dim.4 | Dim.5 | Dim.6 | Dim.7 | Dim.8 | Dim.9 | Dim.10 | Dim.11 | Dim.12 | Dim.13 | Dim.14 | Dim.15 | Dim.16 | Dim.17 | Dim.18 | Dim.19 | Dim.20 | Dim.21 | Dim.22 | Dim.23 | Dim.24 | Dim.25 | Dim.26 | Dim.27 |
|---|---|---|---|---|---|---|---|---|---|---|---|---|---|---|---|---|---|---|---|---|---|---|---|---|---|---|
| 0,63915349 | 0,02858138 | 20,8319863 | 3,14324920 | 3,11513681 | 27,3313440 | 0,00048527 | 16,5783181 | 0,21475250 | 0,63640341 | 0,57156163 | 0,77980175 | 0,13097230 | 0,21613193 | 0,02745399 | 0,14139697 | 0,02330960 | 0,70008372 | 0,12132848 | 0,50046771 | 0,90767914 | 0,05802840 | 0,14223295 | 0,20193463 | 0,08503802 | 0,01754349 | 0,00174594 |
| 42139,0864 | 7524,00158 | 4145,93065 | 1740,33831 | 55165,4735 | 1138,60403 | 0,41816878 | 4,73796363 | 4,34702670 | 0,00464473 | 1,38777002 | 0,81241851 | 1,05164331 | 0,05796750 | 0,00235465 | 0,04264129 | 0,00288195 | 0,00015797 | 0,00002511 | 0,00003898 | 0,00020633 | 0,00001094 | 0,00001417 | 0,00003437 | 0,00000480 | 0,00000538 | 0,00000813 |
| 0,51159973 | 0,00832218 | 11,2463432 | 0,36362631 | 1,74221070 | 17,6684900 | 0,19164231 | 3,19627633 | 0,53639192 | 0,30119235 | 0,01833250 | 0,13558999 | 0,00001542 | 0,12803470 | 0,00904731 | 0,29202424 | 0,13246375 | 0,02898078 | 0,19033320 | 0,12850995 | 0,11122791 | 0,00009948 | 0,35485853 | 0,07889941 | 0,28075225 | 0,03493900 | 0,01160828 |
| 0,22011189 | 0,04713371 | 1,79812207 | 0,05860383 | 0,12254181 | 1,05057008 | 0,05089657 | 0,43291037 | 0,00597063 | 0,01648527 | 0,00085683 | 0,00004340 | 0,02339712 | 0,02000224 | 0,09693642 | 0,02031265 | 0,02545315 | 0,01347320 | 0,00561503 | 0,00142173 | 0,00097844 | 0,00011950 | 0,05452440 | 0,00563505 | 0,00990560 | 0,14700587 | 0,09897092 |
| 0,19077762 | 5,75974819 | 369,532340 | 8,29682377 | 65,4661454 | 526,315162 | 1,53475831 | 162,010982 | 0,35168412 | 13,2016780 | 8,08041847 | 23,5516474 | 0,82995790 | 46,2739784 | 11,2416050 | 21,2646473 | 1,76259479 | 0,49230129 | 0,07975140 | 0,06214091 | 0,10868031 | 0,00008123 | 0,00792440 | 0,00286831 | 0,00456640 | 0,00016498 | 0,00149894 |
| 1,69597345 | 0,13067562 | 14,7697293 | 6,23153641 | 32,8734699 | 4,48990909 | 4,61793301 | 10,4967171 | 3,16459535 | 4,38092590 | 144,743968 | 14,7179965 | 283,660700 | 0,96103888 | 0,57025002 | 0,15187047 | 0,00015190 | 0,04343360 | 0,00050768 | 0,01363220 | 0,00045660 | 0,00041760 | 0,00122538 | 0,00000030 | 0,00005171 | 0,00001517 | 0,00017107 |
| 1093,38462 | 1838,88839 | 63905,3169 | 17983,7563 | 4089,31971 | 9,79558853 | 13,4495082 | 131,589397 | 82,8742809 | 4,19721191 | 10,6641522 | 12,8343856 | 1,45406350 | 1,73160779 | 1,23166220 | 0,05405827 | 0,10908020 | 0,02408579 | 0,00563034 | 0,00961863 | 0,00745168 | 0,00010122 | 0,00017538 | 0,00000184 | 0,00028071 | 0,00002067 | 0,00000279 |
| 141,530150 | 14,5913065 | 84,1953885 | 34,5472614 | 19,0736479 | 3,84148023 | 61,6697094 | 19,0282245 | 0,40282632 | 13,2575192 | 0,78817778 | 70,8405775 | 8,62665082 | 44,4177731 | 33,4023154 | 7,38495248 | 2,19797673 | 0,11685174 | 0,00643595 | 0,01063742 | 0,01992344 | 0,00026530 | 0,00026788 | 0,00022830 | 0,00018250 | 0,00008312 | 0,00010100 |
| 4018,37523 | 112,057398 | 1641,93513 | 505,374748 | 9,32302601 | 66,5776498 | 3720,44088 | 134,989570 | 36,6933038 | 5,62654270 | 9,52601651 | 6,34682468 | 0,06965272 | 1,81672115 | 1,63800730 | 0,00596760 | 0,02591175 | 0,03199704 | 0,00066924 | 0,00000097 | 0,00151760 | 0,00000862 | 0,00012105 | 0,00030197 | 0,00000029 | 0,00000194 | 0,00000007 |
| 0,23072540 | 9,98571580 | 144,404448 | 42,3323563 | 4,89255740 | 0,46354262 | 0,18698120 | 0,89292445 | 0,54050775 | 0,06920558 | 0,15404311 | 0,38980663 | 0,44557670 | 1,43608920 | 0,00947901 | 0,08270628 | 0,00351778 | 0,33895520 | 0,17836850 | 2,18889241 | 0,66106404 | 0,24685734 | 0,05287827 | 0,00370450 | 0,00186650 | 0,01166493 | 0,00740969 |
| 2,37934650 | 6,03006959 | 183,216290 | 33,3187912 | 1,08273410 | 34,3364958 | 15,4704250 | 3,98425761 | 3,59550450 | 0,14749529 | 6,78174690 | 2,32792522 | 5,44758749 | 17,9995839 | 1,47689300 | 3,06307057 | 24,9555501 | 0,33439385 | 0,02152608 | 0,00000075 | 0,00078221 | 0,00056334 | 0,00934435 | 0,00012851 | 0,00038230 | 0,00080562 | 0,00030527 |
| 9,45174999 | 6,42953638 | 381,070364 | 78,2736677 | 21,2117715 | 0,81963062 | 0,15034424 | 0,71272105 | 1,13328853 | 0,18117602 | 0,07860940 | 0,00580819 | 1,68237780 | 0,30132217 | 2,55218610 | 1,69163951 | 1,32002268 | 11,7826139 | 0,40660780 | 0,00024884 | 0,04712620 | 0,01800889 | 0,00109583 | 0,01012052 | 0,00154341 | 0,00177114 | 0,00001732 |
| 2174,18380 | 415,067752 | 6764,41882 | 712,233690 | 2148,71735 | 8893,55994 | 167,101721 | 661,934620 | 28,2137927 | 67,8294325 | 8,89956599 | 0,07720070 | 0,51484281 | 0,51861540 | 0,42023689 | 0,00102368 | 0,06291210 | 0,00294730 | 0,00203057 | 0,00000384 | 0,00000012 | 0,00005664 | 0,00015861 | 0,00000909 | 0,00000000 | 0,00000517 | 0,00000010 |
| 2,32572308 | 0,16656080 | 17,8756911 | 3,05267710 | 5,65751550 | 18,1499730 | 0,91957078 | 5,22460929 | 0,08195999 | 0,00597298 | 0,01360634 | 0,36945007 | 0,02072437 | 0,30444573 | 0,58855532 | 0,48046847 | 0,13109865 | 0,04629920 | 0,51347610 | 0,22256450 | 0,15134611 | 0,16562558 | 0,07787845 | 0,52535608 | 0,07359615 | 0,00322893 | 0,00018961 |
| 210,507400 | 367,037593 | 338,849310 | 13,4229475 | 0,16126990 | 14,6984652 | 250,913221 | 328,298322 | 887,635422 | 1,83126811 | 13,0618638 | 34,4999905 | 4,95561097 | 1,30413985 | 3,78676010 | 1,98040220 | 0,06816109 | 0,02078989 | 0,00078313 | 0,00907645 | 0,00107093 | 0,00000000 | 0,00051675 | 0,00003763 | 0,00007631 | 0,00001435 | 0,00001556 |
| 3,38422110 | 2,90904134 | 612,387904 | 46,6321474 | 231,922622 | 852,185608 | 4,84642031 | 800,703458 | 7,88683014 | 83,3909665 | 4,57583372 | 145,463140 | 0,00495660 | 12,8235915 | 5,63144214 | 8,56101922 | 0,67316288 | 0,08592320 | 0,00460538 | 0,01822513 | 0,00707764 | 0,00090405 | 0,00012533 | 0,00032399 | 0,00001748 | 0,00001439 | 0,00000044 |
| 255,556113 | 430,577229 | 13146,7668 | 2588,93110 | 944,046235 | 2962,18775 | 9,36581487 | 610,890310 | 274,858105 | 5,78724720 | 42,0769828 | 43,1038720 | 4,91065805 | 4,74063737 | 3,82897482 | 0,90450918 | 0,23418010 | 0,01972845 | 0,00000961 | 0,00457794 | 0,00363944 | 0,00294988 | 0,00003628 | 0,00014944 | 0,00104113 | 0,00010167 | 0,00007253 |
| 0,19294592 | 0,01359270 | 8,06589855 | 0,00444477 | 2,43028563 | 10,5703348 | 0,02064639 | 0,73830619 | 0,20399552 | 0,15267987 | 0,17497371 | 0,53937007 | 0,00302915 | 0,80205690 | 0,33947088 | 0,04468990 | 0,01490461 | 0,00015387 | 0,00891090 | 0,18937962 | 0,18250767 | 0,07480768 | 0,07319030 | 0,13703037 | 0,25177297 | 0,09288370 | 0,01201956 |
| 0,43504808 | 0,00000018 | 19,8594972 | 2,35149701 | 14,9160097 | 22,7907503 | 0,00369539 | 9,83978735 | 0,11978457 | 0,32904080 | 0,03399050 | 0,76981400 | 1,10980338 | 2,20980544 | 0,89030195 | 0,31485282 | 0,04444949 | 0,07999100 | 0,13166981 | 0,03789978 | 0,46411540 | 0,19601262 | 0,31680043 | 0,00716925 | 0,04722584 | 0,03815838 | 0,04147314 |
| 15172,7647 | 3047,07685 | 21629,3842 | 71156,3274 | 41,6769704 | 580,117793 | 48,3635470 | 26,7510623 | 4,61077761 | 1,57707337 | 0,19351730 | 0,05392130 | 0,47989027 | 0,18877515 | 0,00303878 | 0,03096652 | 0,00187745 | 0,00012483 | 0,00000327 | 0,00008635 | 0,00000327 | 0,00000013 | 0,00000311 | 0,00000401 | 0,00000180 | 0,00000009 | 0,00000060 |
| 166,555885 | 7,43658990 | 60,1248644 | 23,6418639 | 3,26713300 | 33,9983808 | 14,5324962 | 57,2725758 | 1,10230594 | 1,15614079 | 9,34541449 | 112,931523 | 0,91336055 | 48,5501350 | 13,2899319 | 23,0265730 | 0,03380283 | 0,00000340 | 0,00428201 | 0,00045417 | 0,00464061 | 0,00124592 | 0,00527852 | 0,00010015 | 0,00225760 | 0,00031095 | 0,00045061 |
| 5,05922610 | 0,05190017 | 1,82020091 | 0,21676818 | 3,31542809 | 0,58044543 | 0,00100240 | 0,04552165 | 0,72306754 | 0,11392592 | 1,35441609 | 3,87479372 | 1,82040947 | 0,01580689 | 0,02223790 | 0,04871879 | 0,00160044 | 0,25892035 | 0,30771387 | 1,36560810 | 0,10459928 | 0,82517567 | 0,05506989 | 0,00061600 | 0,00557187 | 0,00343108 | 0,00510380 |
| 897,522190 | 3,53577810 | 77,0819105 | 30,4238321 | 2,78547147 | 61,1066122 | 46,0490017 | 56,0580533 | 30,0859394 | 361,813639 | 136,295014 | 31,6906995 | 47,7640775 | 4,34974648 | 5,43643501 | 1,75360920 | 0,00277050 | 0,00098333 | 0,00369989 | 0,01072842 | 0,00009394 | 0,00000940 | 0,00027430 | 0,00010484 | 0,00127722 | 0,00000101 | 0,00003875 |
| 7320161,79 | 1000742,30 | 100,076255 | 10,6304779 | 386,269632 | 0,65865102 | 0,58882903 | 0,06590395 | 0,05363248 | 0,01049697 | 0,00241213 | 0,01122489 | 0,00056690 | 0,00003520 | 0,00086480 | 0,00016402 | 0,00013690 | 0,00000107 | 0,00000001 | 0,00000060 | 0,00000070 | 0,00000002 | 0,00000000 | 0,00000007 | 0,00000000 | 0,00000000 | 0,00000000 |
| 2535253,33 | 2909836,40 | 210,045035 | 71,4623328 | 11,1453670 | 1,72222163 | 0,53187260 | 0,14556754 | 0,00789829 | 0,00261113 | 0,00029780 | 0,00807742 | 0,00002379 | 0,00027260 | 0,00000930 | 0,00012485 | 0,00000562 | 0,00000390 | 0,00000028 | 0,00000007 | 0,00000040 | 0,00000007 | 0,00000000 | 0,00000000 | 0,00000007 | 0,00000000 | 0,00000000 |
| 17,5524805 | 0,00906952 | 23,9623560 | 0,87906955 | 6,44071102 | 20,9310941 | 0,18186632 | 1,20493891 | 0,02817912 | 0,17536172 | 0,57341234 | 0,55913791 | 0,03129369 | 0,33020754 | 0,05069665 | 0,16629971 | 0,00246505 | 0,67948770 | 3,55633487 | 0,19080073 | 0,26019692 | 0,01609605 | 0,07158813 | 0,04546159 | 0,00418279 | 0,00786329 | 0,00792889 |
| 1153,85571 | 82,5070588 | 1531,95390 | 52,9559870 | 882,061293 | 1819,68568 | 79,4868868 | 4,46094407 | 8,36200614 | 291,121632 | 193,558135 | 6,93476855 | 43,5861510 | 3,00999129 | 0,12269660 | 0,85981390 | 0,63611120 | 0,05822487 | 0,00468635 | 0,00073050 | 0,00136773 | 0,00007510 | 0,00001817 | 0,00054445 | 0,00001680 | 0,00020430 | 0,00006307 |
